# Children’s representations of parents account for multifinality in outcomes of parental control: Evidence from two studies

**DOI:** 10.1017/S0954579425100321

**Published:** 2025-07-02

**Authors:** Haley M. Herbert, Juyoung Kim, Grazyna Kochanska

**Affiliations:** Department of Psychological and Brain Sciences, The University of Iowa, Iowa, USA

**Keywords:** attachment, internal working models, multifinality, parental control

## Abstract

Effects of variations in parents’ control styles, especially the amount of power assertion they deploy, have long been a central question in socialization research. Although severe, harsh control is unanimously considered harmful, research on effects of far more common low-to-moderate power assertion is inconsistent. Drawing from attachment and social cognition traditions, we examined whether children’s representations of parents (Internal Working Models, IWMs) moderated associations between parental power assertion and children’s socialization (violating or embracing rules and values, responsiveness to parents). In two studies of community families (Family Study, FS, *N* = 102, and Children and Parents Study, CAPS, *N* = 200), employing observations and reports, we assessed parental power assertion at age 4.5, children’s IWMs at ages 8 in FS and 4.5 in CAPS, and socialization outcomes at ages 10 and 12 in FS and 4.5 in CAPS. In FS, children’s IWMs of the parent moderated effects of parental power assertion on socialization outcomes in mother- and father-child dyads (*β*s = 0.47, 0.41, respectively): Power assertion had detrimental effects only for children with negative IWMs of their parents. In CAPS, findings were replicated for mother-child dyads (*β* = 0.24). We highlight origins of multifinality in socialization sequelae of parental control.

## Introduction

Why do some children embark on adaptive paths toward prosocial, rule-abiding conduct, and robust social competence, and willingly embrace and internalize parents’ socialization messages, whereas others enter maladaptive paths toward callousness, disregard for conduct rules, disruptive and antisocial behavior, and impoverished competence, and reject parental socialization influence? Understanding origins of multifinality in trajectories of children’s outcomes has long been developmental psychopathology’s key aim. Given the profound burdens for individuals, families, and the society associated with disruptive, antisocial trajectories, elucidating factors that account for such divergent developmental paths remains a fundamental broad goal in developmental psychology and psychopathology.

Voluminous literature has focused on how individual differences in parenting predict children’s socialization outcomes. The construct of control, and especially the degree of power assertion parents deploy to influence children’s behavior, has been a key aspect of parenting in almost all influential theories (Baldwin, 1955; Becker, 1964; Grolnick & Pomerantz, 2009; Hoffman, 1983; Lansford, 2022; Maccoby & Martin, 1983; Schaefer, 1959; Sears et al., 1957). Highly power-assertive, harsh control is universally considered detrimental to development; however, effects of milder power assertion, commonly used by essentially all parents of young children (Vittrup et al., 2006), and unavoidable while regulating children’s behavior, continue to be a subject of intense debate (e.g., Baumrind et al., 2002; Gershoff, 2002b; Gershoff et al., 2019; Larzelere et al., 2019; Larzelere & Kuhn, 2005). The mixed evidence has inspired a search for moderators of effects of parental power assertion.

Bowlby’s pioneering and still flourishing attachment theory (1969/1982) – and generally, theories of early relationships (Humphreys et al., 2024; L. A. Sroufe, 2005; Thompson, 2015; Thompson, 2021) – have promise to inform this search. Attachment, an evolutionarily based proximity-regulating biobehavioral system, provides the child with confidence in protection and supports behavioral, emotional, and physiological regulation of threat and distress. That view has been expanded to include attachment’s role in socialization: A secure attachment engenders child receptiveness and willingness to embrace parental socialization (Goffin et al., 2018; van IJzendoorn, 1997; Kochanska et al., 2015; Laible & Thompson, 2007). Perhaps even more importantly, security can moderate future socialization processes unfolding in the parent-child dyad, including parent-child control that comes “online” in the second year and rapidly increases in early childhood and beyond (Kochanska et al., 2019; Kochanska & An, 2024b; L. A. Sroufe, 2005; Thompson, 2006).

Growing evidence has supported this view. Across multiple studies, power-assertive control, used in daily interactions to regulate child behavior, has been indeed detrimental to children’s socialization outcomes, but *only* in parent-child dyads that had been insecure in infancy (Bendel-Stenzel et al., 2023; Kochanska & An, 2024a,b; Kochanska et al., 2019; Kochanska & Kim, 2012). Security has effectively counteracted negative impacts of parental control.

What can account for this striking pattern of multifinality? We have proposed that Bowlby’s central construct of children’s representations of the parents, or Internal Working Models (IWMs), emerging in the context of early attachment and reflecting their relational experience, may be key to understanding those findings (Kochanska et al., 2019; Kochanska & An, 2024a,b; Kochanska & Kim, 2012).

In insecure relationships, children’s IWMs are characterized by views of the parent as negative, hostile, unfair, untrustworthy, and unresponsive. Those IWMs then bias children’s perception of parental control. Children with negative IWMs of parents see control as hostile, arbitrary, and mean-spirited, even if power is mild. The child comes to resent and reject parental socialization influence and messages (Gershoff, 2002a; Grusec & Goodnow, 1994).

But in secure relationships, children view their parents as trustworthy, responsive, and accepting (Bowlby, 1969/1982; Bretherton & Munholland, 2008; Carlson et al., 2004; Cassidy et al., 2013; Dykas & Cassidy, 2011; L. A. Sroufe, 2016; Thompson, 2021), and view control – even if firm and assertive – as well intentioned. They willingly embrace and cooperate with the parent, embarking on a positive socialization path (Bugental & Johnston, 2000; Grusec & Goodnow, 1994; Kochanska et al., 2019, 2010; Y-E. Lee et al., 2016; Maccoby, 2007; Toth et al., 1997).

Empirical evidence, although motivated by divergent traditions, appears consistent with such a model. Rohner, from the perspective of interpersonal acceptance–rejection theory (Rohner & Lansford, 2017) argued that punishments and other forms of power assertion have detrimental effects only if children perceive their parents as rejecting, but not when they see them as accepting (Rohner & Melendez-Rhodes, 2019). Many researchers have focused on warmth, a construct related to security, and they pointed out that detrimental effects of power assertion are reduced in parent-child relationships characterized by warmth. Parke (1969), from the early learning perspective, showed that punishment by a warm agent was more effective than that by an aloof agent. In Baumrind’s tradition (1971), moderate power assertion is a component of the optimal authoritative style when accompanied by warmth. Several studies have reported similar findings (Darling & Steinberg, 1993; Deater-Deckard et al., 2006; Kim & Kochanska, 2015; McKee et al., 2007), but others have failed to replicate them (e.g., S. J. Lee et al., 2013; Stacks et al., 2009) or found that relations depended on the studied outcome and culture (Lansford et al., 2014). Some research was inconclusive (Lansford, 2022;S. J. Lee et al., 2013; Ward et al., 2020; Wiggers & Paas, 2022; S. J. Lee et al., 2013; Ward et al., 2020).

Many of those studies have relied on measures of children’s reports of warmth, often retrospective, rather than on assessments of their IWMs of parents informed by attachment theory. Further, little is known about the processes involved in mother- vs. father-child dyads. Kochanska and An (2024a) reported that maternal power assertion at 16 months was associated with higher mother-rated child disruptive behavior at age 3, but *only* for children whose IWMs of their mothers, assessed with attachment-informed story stems techniques, were negative. Positive IWMs buffered those effects. There were no findings for fathers.

We now continue and considerably expand Kochanska and An’s (2024a) investigation. We report findings from two longitudinal studies of typical, low-risk families: an earlier Family Study (FS) and the ongoing Children and Parents Study (CAPS), from the same community in U.S. Midwest. Children’s IWMs of parents were assessed in middle childhood in FS, using children’s explicit reports of the parent as safe haven and secure base, and at preschool age in CAPS, using semi-projective play narratives. The measures of children’s socialization outcomes were assessed twice in early preadolescence in FS, using children’s and parents’ reports and observations, and at preschool age in CAPS, using observations. In each study, all measures were parallel for mother- and father-child dyads, to examine similarities and differences and address the stubborn gap in knowledge persistent in the social-emotional development field (Cabrera et al., 2018), and to explore potential replications across the two relationships.

### Relevance to developmental psychopathology

We explicitly sought to incorporate recently articulated priorities for future research in developmental psychopathology (Pollak, 2024; Special Issue, *Development and Psychopathology*). In the Special Issue, several scholars argued for expanding our focus from that on largely negative developmental outcomes to include also multiple aspects of children’s positive development (Eisenberg et al., 2024; Pluess, 2024). We examined both negative and positive socialization outcomes (e.g., violating parents’ and other adults’ rules and values, but also embracing the rules, prosociality, receptiveness to parents’ requests and cues).

Further, we considered early parent-child relationship as foundational, or “a cornerstone” for future developmental trajectories (Humphreys et al., 2024) and key for children’s emerging mindsets (Dodge, 2024). We studied children’s IWMs of the parents, presumably formed in early relationships, as one important factor that accounts for presence or absence of detrimental effects of parental power-assertive control (Kochanska & An, 2024b).

We remained mindful of the emphasis on replications in psychology. Replicating interaction effects is especially important (Rutter & Pickles, 1991), but also challenging. As researchers from Open Science Collaboration (2015) stated (p. aac4716–5): “Among original, significant effects, 23 of the 49 (47%) that tested main or simple effects replicated at *p* < 0.05, but just 8 of the 37 (22%) that tested interaction effects did.”

In developmental psychology and psychopathology, those challenges are unique and perhaps greater, with “replication” having several meanings. The difficulty is due to at least three reasons. One, few laboratories have longitudinal data from several samples, collected using comparable methods. Two, we often aim to replicate interaction effects at different points in development; thus, we naturally tailor our measures to children’s ages. The absence of replication may not invalidate the studied effect, but rather, it may indicate that it is age specific. Three, we often explore if interactions replicate across mother- and father-child dyads. If they do not – then an absence of replication is informative in and of itself, as it might indicate different processes operating in the two relationships – a poorly understood issue.

In this work, our replication effort represents a “varied replication,” highly valued in and appropriate for developmental psychopathology research (van IJzendoorn & Bakermans-Kranenburg, 2024). Although the theoretical conceptualization of the interaction effect being replicated was essentially the same in both studies, several empirical aspects were different. The groups of parents and children in FS and CAPS came from different cohorts, separated by 16–17 years. Although we kept measures as similar as possible, they were necessarily tailored to children’s ages: FS covered longitudinal age span from preschool age to early preadolescence, and CAPS data were concurrent at preschool age. In FS, children’s IWMs reflected their explicit representations of the parent and in CAPS, they reflected implicit representations. The measures of socialization outcomes likewise reflected age-appropriate constructs. In each study, we examined the processes in mother- and father-child dyads. The varied replication design affords confidence in effects replicated across studies.

### Family Study (FS)

#### Method

##### Participants.

This longitudinal study involved 102 two-parent community families from a college town, a small city, and rural areas in the Midwest (mothers, fathers, and infants). Parents of typically developing infants, biological children, most born in 2001, volunteered in response to broadly distributed advertisements. The families were mostly White, but 20% of them (*N* = 20) included one or both non-White parents (demographic details are in Supplement 1, FS: Table S1). The University of Iowa approved the study (Developmental Pathways to Antisocial Behavior: A Translational Research Program, 200107049). The parents completed informed consents at the outset, and the children completed assents at the age of 7 years.

##### Overview of design.

We report data collected at 52 months (age 4.5, *N* = 99, 49 girls), 100 months (age 8, *N* = 87, 41 girls), 122 months (age 10, *N* = 82, 37 girls), and 147 months (age 12, *N* = 79, 37 girls). At each time, female experimenters (Es) conducted two 2- to 4-hr sessions (video-recorded), one with each parent, in our laboratory (at age 8, there was one session, with no parent–child observations, and the assessments focused on the child). The sessions encompassed multiple naturalistic but standard paradigms and contexts. The laboratory included a naturalistically furnished Living Room and a Play Room. Multiple teams coded behavioral data (generally, the same coder coded the child with only one parent). Between 15 and 20% of cases were sampled for reliability, with frequent realignments. There were no significant differences in any assessed construct between families that returned at age 12 and those that did not.

### Measures

#### Mothers’ and fathers’ power-assertive control, age 4.5 years

The parent and child were observed during several naturalistic, scripted control contexts (75 min with each parent), which encompassed a “Do” context, with the child asked to perform a desired behavior (toy cleanup, 10 min), and “Don’t” contexts, with the child asked to refrain from prohibited behavior (touching very attractive, easily reachable objects, designated by the parent as off limits, 65 min). Parental control was coded for every 30-s segment (inter-coder reliability, kappas, were .68 to .94; details of coding and data aggregation, resulting in the overall power assertion score for each parent, are in Supplement 2, FS: Coding and Data Aggregation).

#### Children’s self-reported IWMs of parents, age 8 years

Children were interviewed using Kerns Security Scale (KSS; Brumariu et al., 2018; Kerns et al., 1996), a well-validated 15-item questionnaire. KSS can be legitimately considered a measure of the child’s explicit IWM of the parent in terms of perceived trust and expected responsiveness and availability. E read the questionnaire to the child without the parent present, and the child indicated, first, which description of each item was most like him or her, and second, whether this description was *very true* or *sort of true*. Each item was scored from 1 to 4 (higher scores indicate a more positive IWM). The scores were tallied. Cronbach’s alphas were .67 and .68, for children’s perceptions of the mothers and fathers, respectively.

#### Children’s self-reported internalization of adult values, ages 10 and 12 years

At both ages, children completed a slightly adapted Adolescent Values Inventory (Allen et al., 1989), selecting a subset of 12 items, rated from 1 to 4, representing embrace of adult values (Allen et al., 1989; e.g., “Some kids think a kid who smokes cigarettes is cool but other kids don’t respect a kid who smokes cigarettes”). Those were averaged into one score at each age (Cronbach’s alphas were .69 and .64 at ages 10 and 12, respectively), and then across the two ages, *r*(71) = .39, *p* < .001.

#### Children’s parent-reported prosociality, ages 10 and 12 years

At both ages, mothers and fathers completed MacArthur Health Behavior Questionnaire (Essex et al., 2002). We selected the Prosocial Behavior scale that targets children’s helpful, empathic, cooperative conduct (20 items, rated from 1 = *rarely applies* to 3 = *certainly applies*). Cronbach’s alphas for mothers and fathers were .90 and .92 at age 10 and .90 and .90 at age 12. The ratings correlated across the two assessments, for mothers, *r*(76) = .75, and for fathers, *r*(73) = .73, *ps* < .001, and were averaged across both ages for each parent.

#### Children’s observed responsiveness to parents, ages 10 and 12

At each age, children were observed interacting with their parents in standard contexts (total of 81 min for a child with each parent; 15 contexts at age 10 and 14 at age 12), adapted from attachment-informed research programs (L. A. Sroufe et al., 2005; Allen et al., 2003; for details, see Bendel-Stenzel et al., 2023; Boldt et al., 2016). The contexts included discussions of difficult, troubling, or controversial issues, “hot topics” (conflicts), fun issues, and interactions involving puzzles or a snack. Child responsiveness coding, ranging from 1 = *highly unresponsive* to 7 = *highly responsive*, incorporated the child’s sensitivity (detection, interpretation, and prompt, appropriate, and contingent response to the parent’s cues, signals, or overtures, etc.), acceptance (warmth, enjoyment, affection, resentment toward the parent), and cooperation (respect for the parent, acknowledging his/her attempts). Inter-coder reliability, weighted kappas, were .74 to .91; details of coding and data aggregation, resulting in the child’s score with each parent, are in Supplement 2, FS: Coding and Data Aggregation.

## Results

### Preliminary analyses

All descriptive data and correlations are in Table 1. Higher parental power-assertive control at age 4.5 was associated with children’s lower scores on positive socialization outcomes at ages 10 – 12 in both dyads, with one exception of maternal power-assertive control and mother-rated prosociality. Children with more positive IWMs of their fathers at age 8 were more responsive to them at ages 10 – 12. Child socialization outcomes correlated with each other. All constructs assessed for both mother- and father-child dyads had significant cross-parent correlations.

#### Children’s Internal Working Model as a moderator of the relation between parental power-assertive control and children’s socialization outcomes

We estimated moderation models separately in mother-child and father-child dyads. Parental power-assertive control at age 4.5 and children’s IWMs at age 8 were modeled as the predictor and moderator, respectively. For easy interpretation of the results, we mean-centered children’s IWM. Parental power-assertive control variables were not mean-centered because they were standardized scores. A latent variable of children’s socialization outcomes with three indicators (i.e., internalization of adult values, prosociality, and responsiveness to the parent) at ages 10 – 12 was modeled as the outcome. Children’s gender was covaried. Further, because the dynamics between the child and the other parent may affect those between the child and the target parent, the other parent’s power-assertive control was also controlled.

The models were tested in Mplus 7 (Muthén & Muthén, 1998–2012) with the full information maximum likelihood estimator to handle missing data. Model fit is considered good when the comparative fit index (CFI) is larger than or equal to .95 and the root mean square error of approximation (RMSEA) is less than or equal to .05 and acceptable when CFI is larger than or equal to .90 and RMSEA is less than or equal to .08 (Hu & Bentler, 1999; Little, 2013). For significant moderation, we probed and plotted simple slopes at 1 standard deviation (*SD*) above and below the mean (Aiken & West, 1991).

#### Mother-child dyads

**Figure 1.**
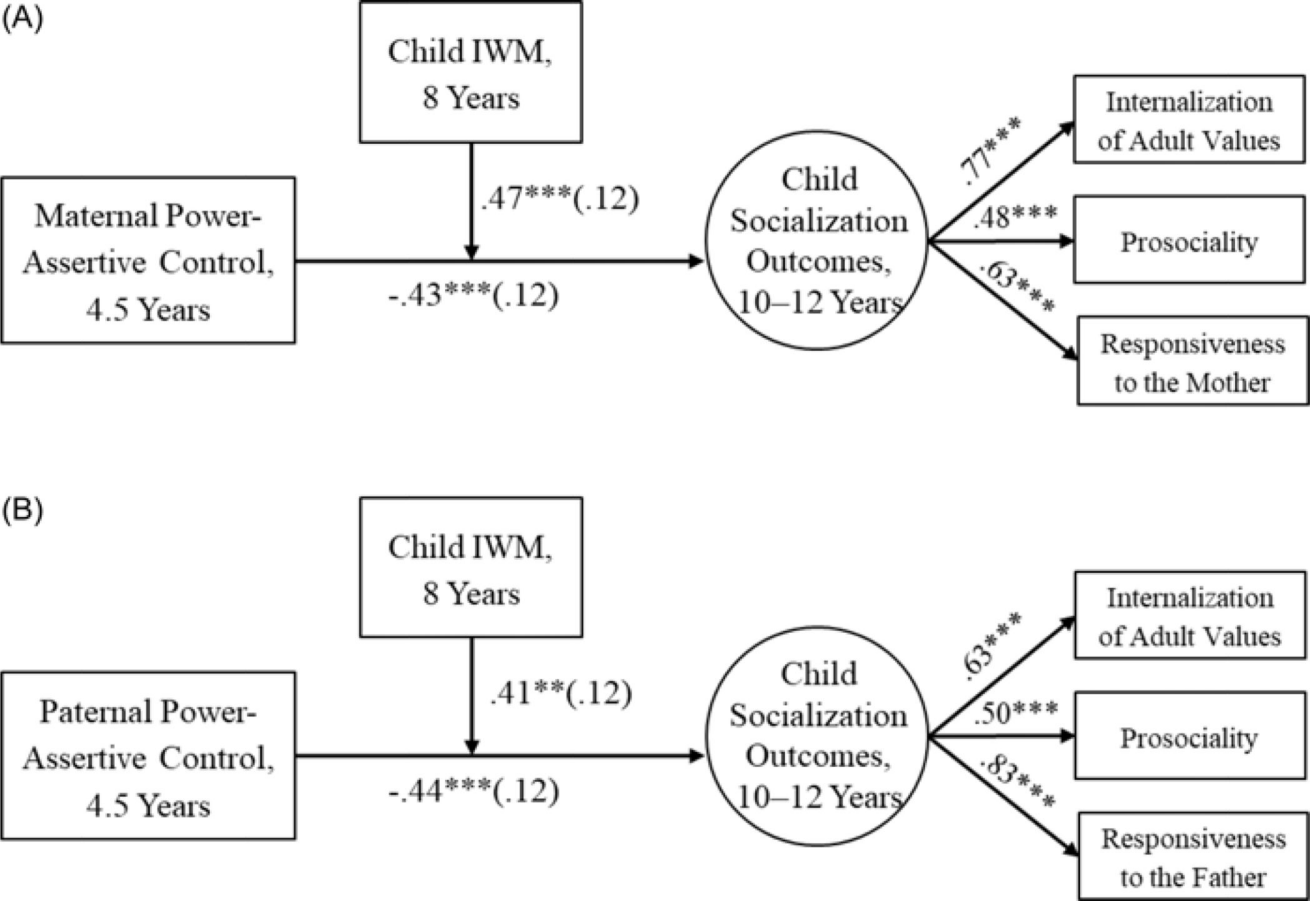
Family Study: Relations between early parental power-assertive control and future children’s socialization outcomes moderated by children’s Internal Working Models of the parents. *Note*: *A* = mother-child dyads. *B* = father-child dyads. IWM = Internal Working Model. Standardized loadings and coefficients (standard errors in parentheses) are presented. Child gender and the other parent’s power assertion were covaried but not depicted in the figures for clarity. **p < .01. ***p < .001.

**Figure 2.**
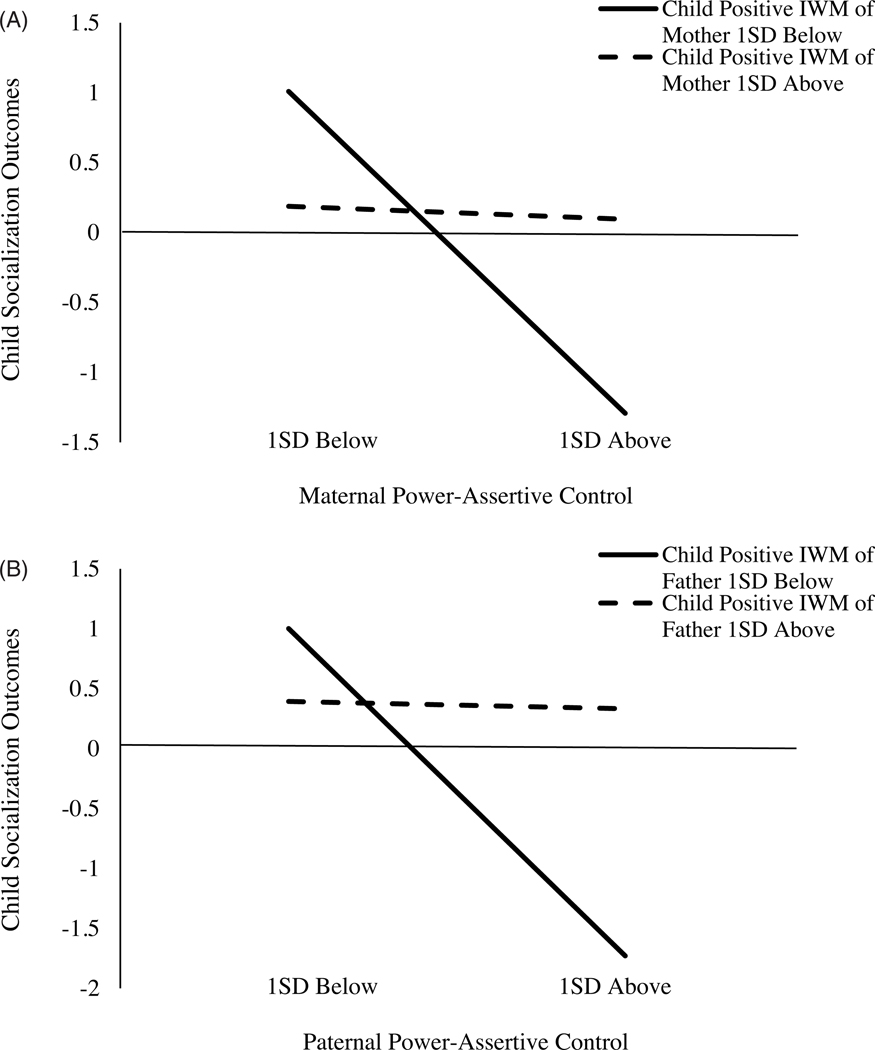
Family Study: Children’s Internal Working Models of the parents moderate the relation between parental power-assertive control and children’s socialization outcomes. *Note*: *A* = mother-child dyads. *B* = father-child dyads. IWM = Internal Working Model. A simple slope at 1 SD below was significant at *p* < .001 in both dyads.

The results are presented in Figure 1A. The final model fit was acceptable, CFI = .92, RMSEA = .07 with 90% confidence interval (CI) [.00, .13]. Maternal power-assertive control at age 4.5 was negatively related to child socialization outcomes at ages 10 – 12. This relation was moderated by children’s IWMs of their mothers at age 8. Higher maternal power-assertive control at preschool age was associated with less positive child socialization outcomes in preadolescence only for children with less positive IWMs of their mothers, *B* = −1.41, *SE* = 0.38, *p* < .001, 95% CI [−2.14, −0.67], but not for children with more positive IWMs of their mothers (Figure 2A).

#### Father-child dyads

The model fit the data well, CFI = .96, RMSEA = .05 [.00, .11], and the results from mother-child dyads were robustly replicated in father-child dyads (Figure 1B). Paternal power-assertive control at age 4.5 was negatively related to child socialization outcomes at ages 10 – 12 years, but it was moderated by children’s IWMs of the fathers. Higher paternal power-assertive control was related to less positive child future socialization outcomes only for children with less positive IWM of the fathers, *B* = −1.63, SE = 0.41, *p* < .001, 95% CI [−2.43, −0.82], but not for children with more positive IWMs of the fathers (Figure 2B).

### Children and Parents Study (CAPS)

#### Method

##### Participants.

Two hundred two-parent community families with infants (born in 2017 and 2018; 96 girls), from the same general area as the FS participants, volunteered for the study. They were mostly White, but in 20% of families (*N* = 40), one or both parents were not “White Alone,” i.e., they reported ethnicity as Latino and/or race as non-White (demographic details are in Supplement 3, CAPS: Table S2). The University of Iowa IRB approved the study (CAPS, 201701705); the parents completed informed consents at the entry to the study.

##### Overview of design.

The data reported in this article were collected when children were 8 months (*N* = 200, 96 girls) and 52 months (age 4.5, *N* = 177, 86 girls; attrition was due to the concurrent COVID-19 pandemic, and *N* includes also families that completed online measures only). At 8 months, there was a 2-hr home session (half with each parent); at 4.5 years, each parent-child dyad participated in a 2.5–3.5-hr laboratory session. As in FS, the sessions (video-recorded) encompassed a broad range of paradigms and contexts; the laboratory sessions were conducted in the same physical setting as FS. Our overall approach to coding was comparable to FS.

Children’s IWMs of the child, parental power assertion, and children’s socialization outcomes were all observed at age 4.5. Because the two latter sets of measures were concurrent, we additionally included a covariate (child difficulty, operationalized as anger proneness, obtained at age 8 months), to reduce unmeasured bias due to some of their shared variance.

### Measures

#### Mothers’ and fathers’ power-assertive control, age 4.5 years

The coding and data reduction, although essentially parallel to FS, were slightly adapted and simplified, due to logistical constraints (note that this resulted in a different metric than FS). Each parent’s power assertion was observed during 10-min toy cleanup (coded for every 30-s segment) and 15 min of other interactions (e.g., introduction to the lab, snack; coded for every 20-s segment). In the toy cleanup, parental control revolved around the issue of picking up toys; in the other interactions, it could involve various issues (inter-coder reliability, weighted kappas, were .61 to .92; details of coding and data reduction, resulting in one power assertive score for each parent, are in Supplement 4, CAPS: Coding and Data Aggregation).

#### Children’s IWMs of parents, age 4.5 years

E presented the child with six stories (three for each parent depicted as the protagonist). Small doll figures and props (furniture, dishes, trees, etc.) were used in all stories. The stories (Lemonade pitcher/Hot mac and cheese; Skateboard/Swing, Bat in bedroom/Spider in bathroom, and a warmup story, Birthday Party, not coded), originally inspired by MacArthur Story Stems Battery (MSSB; Bretherton et al., 1990; Buchsbaum & Emde, 1990; Holmberg et al., 2007; Oppenheim et al., 1997), were further adapted from Davies et al. (2018). In each, the parent issued a directive (e.g., “don’t touch the hot food”); the child disobeyed and was hurt. Having presented the story stem, *E*, using a standard set of probes, asked the child to show and tell what happened next. Inter-coder reliability, weighted kappas, were .81 to .92; details of coding and data reduction, resulting in the score of the child’s Positive Representation of each parent, are in Supplement 4, CAPS: Coding and Data Aggregation.

#### Children’s violations of parental prohibition, 4.5 years

Upon the entry to the lab’s Living Room, E pointed out a low shelf with multiple attractive objects and asked the parent to convey to the child that those were off limits and to enforce the prohibition throughout the session. Close to the end of the session, E brought in a tray with blocks, set it in front of the shelf, and asked the parent to remind the child of the prohibition and to request that the child sort the blocks on the tray. Then the parent moved to the Play Room, and the child remained alone in the Living Room for the next 8 min.

##### Coding:

Child behavior was coded for each 5-s interval. For this report, we selected Brief Touch = *touching object(s) on the shelf for less than 3 s*, and Long Touch = *touching object(s) on the shelf for 3 s or more*. Inter-coder reliability, kappas, ranged from .81 to .87.

##### Data reduction:

The instances of each of the two codes that occurred while the child remained in the Living Room (children occasionally left the room) were tallied and divided by the number of 5-s segments spent in the Living Room. These two scores were then summed.

#### Children’s observed rule-compatible conduct, age 4.5 years

##### Observed context:

E brought in a basket with stuffed animals and invited the child to play a game to win a prize. To win, the child had to guess what animals were hidden under scarves (Aksan & Kochanska, 2005). E asked the child, gently but seriously, to follow the rules (not peeking under a scarf or in the basket and touching only with the tip of one finger). The child was then left alone for 3 min. The game was impossible to win if the rules were followed. Upon return, E apologized for using “wrong animals” and the child played again, using an easy-to-guess animal, until they won a prize. Child behavior was coded for each 3-s segment as rule compatible or violating one or more rules (inter-coder reliability, kappas, were .78 – 1.00; details of coding and data reduction, resulting in one score of rule-compatible conduct, are in Supplement 4, CAPS: Coding and Data Aggregation).

#### Children’s responsiveness to parents, age 4.5 years

##### Observed contexts, coding, and data reduction:

The approach was essentially parallel to FS (with minor adaptations, which resulted in a different metric). Child responsiveness was coded in three 5-min contexts (introduction to the room, waiting for snack, and play; coded for each 1-min segment). The codes were added for each context. The codes ranged from 1 = *not responsive* to 5 = *highly responsive*, with the definition of responsiveness the same as in FS. Inter-coder reliability, weighted kappas, were .60 to .88. The three scores were then averaged across the contexts into an overall responsiveness score for the child (with each parent).

### Covariates

Children’s gender was covaried. As in FS, we controlled for the other parent’s power assertive control. As aforementioned, we also covaried the child’s proneness to anger at 8 months, coded in the 60-s Car Seat episode from Laboratory Temperament Assessment Battery (Goldsmith & Rothbart, 1996). For each 5 s, coders rated children’s body anger (0 = *none*, to 4 = *strong*), facial anger (0 = *none*, to 3 = *strong*), and vocal anger (0 = *none*, to 3 = *strong*), and latency to express anger (inter-coder reliability, kappas, were .68 to .87; ICC .99). The final composite was an average of standardized body, facial, and vocal anger scores and reversed latency. Cronbach’s alpha was .80.

## Results

### Preliminary analyses

All descriptive data and correlations are in Table 2. Higher power-assertive control was associated with less positive child socialization for all three outcomes in mother-child dyads (and with children’s less positive IWM of the mother). In father-child dyads, however, higher paternal power-assertive control was associated only with one outcome – more violations of paternal prohibition. Child socialization outcomes correlated with each other, with one exception (i.e., child violation of paternal prohibition and child responsiveness to the father). All constructs obtained for both mother- and father-child dyads were significantly correlated across the dyads.

### Children’s Internal Working Model as a moderator of the relation between parental power-assertive control and children’s socialization outcomes

The models were estimated parallel to FS. Parental power-assertive control and children’s IWMs were standardized and modeled as the predictor and the moderator, respectively. A latent variable of children’s socialization outcomes with three indicators (i.e., violations of parental prohibition, rule-compatible conduct, and responsiveness to the parent) was modeled as the outcome in mother-child dyads. In father-child dyads, however, we used three separate observed outcomes because the indicator of responsiveness to the father did not load onto the latent variable. Children’s gender, anger proneness at 8 months, and the other parent’s power-assertive control were the covariates.

### Mother-child dyads

**Figure 3.**
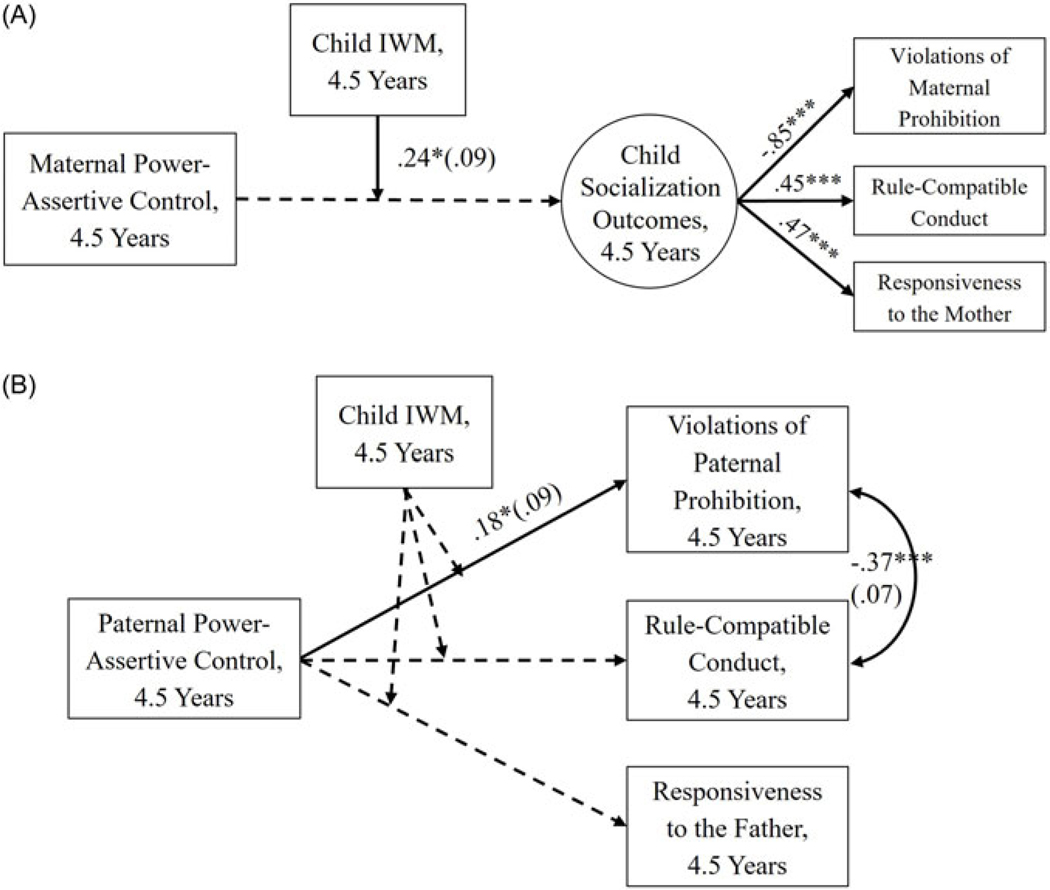
Children and Parents Study: Relation between parental power-assertive control and children’s socialization outcomes moderated by children’s Internal Working Models of the parents. *Note: A* = mother-child dyads. *B* = father-child dyads. IWM = Internal Working Model. Standardized loadings and coefficients (standard errors in parentheses) for significant paths are presented. A dashed line represents a non-significant path. Child gender, the other parent’s power assertion, and child anger proneness at 8 months were covaried but not depicted in the figure for clarity. *p < .05. ***p < .001.

**Figure 4.**
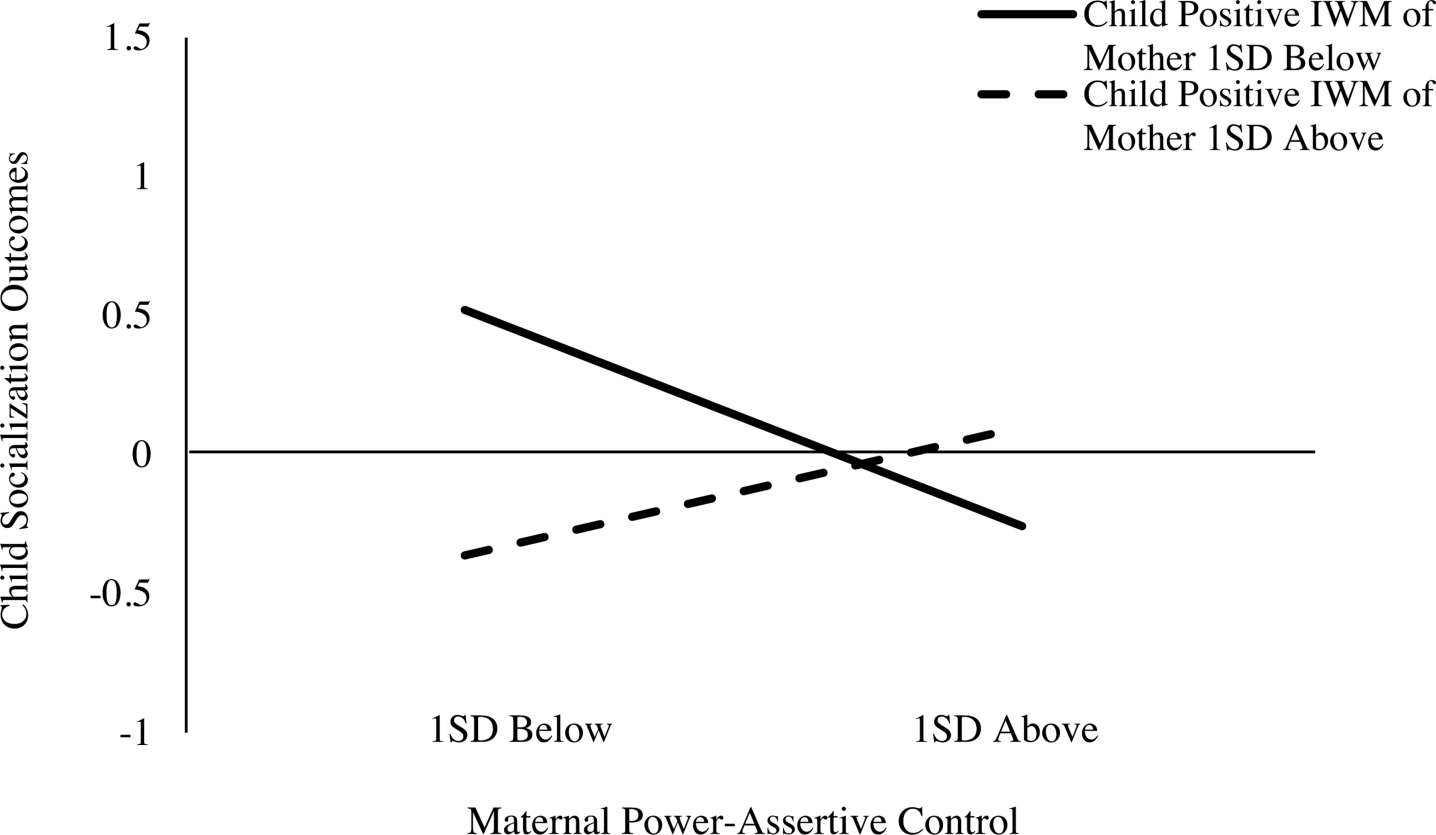
Children and Parents Study: Children’s Internal Working Models of the mothers moderate the relation between maternal power-assertive control and children’s socialization outcomes. *Note:* IWM = Internal Working Model. A simple slope at 1 SD below was significant at *p* < .05.

The final model fit the data well, CFI = .95, RMSEA = .05 [.00, .08], and the results are presented in Figure 3A. The results were consistent with FS. The relation between maternal power-assertive control and child socialization outcomes was significant and negative, and this relation was moderated by child IWMs. Higher maternal power-assertive control was associated with poorer child socialization outcomes for children with less positive IWMs of their mothers, *B* = −0.46, *SE* = 0.22, *p* < .05, 95% CI [−0.89, −0.03], but not for children with more positive IWMs of the mothers (Figure 4).

### Father-child dyads

The final model fit was good, CFI = 1.00, RMSEA = .00 [.00, .08]. The results are in Figure 3B. Among the relations between paternal power-assertive control and child socialization outcomes, only one was significant: Higher paternal power-assertive control was related to more violations of paternal prohibition. In contrast to mother-child dyads, we found no evidence of moderation by the child’s IWM of their fathers.

## General discussion

The two studies together illustrate the rewards – and challenges – of “varied replications” involving interactions in developmental research (van IJzendoorn & Bakermans-Kranenburg, 2024). Because the age ranges studied in FS and CAPS were so different, naturally many measures were not directly comparable. Additionally, because FS began almost two decades before CAPS, their theoretical frameworks only partially overlapped, with our focus on children’s IWMs expanding and evolving over time. When we were conducting FS, we did not administer semi-projective measures of children’s IWMs of their parents; however, we did use the well-established KSS, which can be legitimately viewed as a measure of the child’s perception of the parent as safe haven and secure base. The more recent CAPS was explicitly designed to target children’s IWMs as key constructs, assessed with the attachment-informed MSSB-based measure.

Despite such challenges, we replicated significant and robust moderating effects of children’s IWMs on the relations between maternal power assertion and socialization outcomes for mothers and children across both studies, FS and CAPS (varied replication). Within the studies, we replicated the effects across mother- and father-child dyads in FS (exact replication), but not in CAPS (where the findings were only for mothers and children). Below, we discuss these findings and summarize issues in the replication of conceptualization and measurement across two studies, followed by alternative explanations and future directions.

### Children’s IWMs of the parents as moderators of effects of parental power assertion

Perhaps the strongest pattern of results apparent in both studies involves the key component of our theoretical model: Children’s IWMs as moderators of the effects of power assertion. In most extant studies, children’s IWMs have been explored either as *products* of the child’s early relational experiences and/or as *predictors* of children’s social and emotional functioning (Kerns & Seibert, 2021; Thompson, 2021). In the current work, we adopted a different lens: We examined the rarely studied role of IWMs as *moderators of parental control influence* (Kochanska et al., 2019; Kochanska & An, 2024a, b).

The findings were most impressive for mothers and children, across FS and CAPS. Despite the differences in measures and ages, we supported our hypothesis that differences in children’s IWMs of their mother account for the multifinality of effects of maternal power assertion on socialization outcomes in preadolescence (FS) and at preschool age (CAPS). Power assertion was detrimental only for children who perceived their parents as relatively unresponsive, untrustworthy, and unavailable. Additionally, the model was also robustly supported for father-child dyads in FS.

Although in this work we focused specifically on effects of parental power-assertive discipline on children’s outcomes, our findings fit well with broader perspectives on development that have increasingly emphasized the key importance of children’s subjective construals of their experiences rather than of “objective” parameters of those experiences when examining developmental outcomes (Smith & Pollak, 2021; L. A. Sroufe & J. Sroufe, 2025). In the current work, whether or not the child perceived or represented their parent as reliably trustworthy, available, competent, and supportive was a decisive factor determining whether parental control would or would not undermine future socialization outcomes.

Our findings of differences between mother- and father-child dyads in CAPS (but not FS) add to the limited but growing evidence of differences in socialization processes in the two relationships. Research on fathers’ parenting has been on the rise (Cabrera et al., 2018; Volling et al., 2019; Volling & Palkovitz, 2021). But as evidence accumulates, so do the challenges to explain the findings.

As aforementioned, differences in processes in mother- and father-child relationships add to the challenges involved in replications of effects in longitudinal trajectories, when “parent gender” is the distinct feature and the replication targets the studied process in both relationships. How should we interpret finding expected effects only in one of the two relationships – as a replication failure or as an insight into differences in the two relationships? Our pattern of findings in CAPS indicates that perhaps at a young age, the child’s representation of the mother plays a more potent role than that of the father; however, by middle childhood, both are equally influential, consistent with the findings in FS. Such a working hypothesis is consistent with a meta-analysis of effects of paternal sensitivity that indicated stronger effects for older children (Rodrigues et al., 2021).

We note that interpreting the lack of replication in CAPS of the key moderating effect of children’s IWMs for fathers and children, found in FS, is challenging from a statistical point of view. Recall that in FS, we examined the moderating effect of child IWM of the father on the link between paternal power assertion and the latent variable of child socialization outcome. But in CAPS, we were unable to recover a latent factor for socialization outcomes in father-child dyads. However, we were able to examine the three moderation effects: Child IWM moderating the links between paternal power assertion and child violations of prohibition, rule-compatible conduct, and responsiveness to the father. The review of effect sizes (Betas) was certainly persuasive. In FS, Beta was .41 (SE = .12), *p* = .001. In CAPS, Betas for child violations of prohibition, rule-compatible conduct, and responsiveness to the father were .02 (SE = .08), −.04 (SE = .08), and −.15 (SE = .08), respectively (none were significant).

### Conceptualization and measurement of children’s IWMs of their parents

In FS, children’s IWMs were assessed using children’s explicit perceptions of their parents (as a cautionary note, although Kerns Security Scale is broadly used, its internal consistency in FS was relatively modest). In CAPS, children’s IWMs were measured in a more implicit way, using semi-projective narratives. Although children’s play had been long considered a source of information about their inner worlds, contemporary attachment-informed research can be traced to MSSB (Bretherton et al., 1990; Buchsbaum & Emde, 1990; Buchsbaum et al., 1992). Since then, this approach has been adapted and often modified by multiple teams, resulting in carefully coded observational material providing useful insights into young children’s representations of their attachment figures and relationships (e.g., Bretherton et al., 1990; Cassidy, 1988; Davies et al, 2018; Granot& Mayseless, 2001; Kerns & Seibert, 2021; Kochanska & An, 2024a; Moss et al., 2009; Oppenheim et al., 1997; Toth et al., 1997, 2002). The number and content of the story stems and the coding varies across laboratories, depending on their research goals. Although more nuanced coding has been used, a simpler coding of the child’s positivity or negativity of their representation of the parent, which we adopted, is common (e.g., Davies et al., 2018; Toth et al., 1997).

Whether the explicit and semi-projective measures of IWMs cohere is an important question. In a recent review, Kerns and Seibert (2021) reported modest evidence of coherence in middle childhood and emphasized that more research is needed.

### Conceptualization and measurement of socialization outcomes

Embracing the recently proposed focus in developmental psychopathology to include positive developmental outcomes along with the negative ones (Eisenberg et al., 2024; Pluess, 2024) we sought to target children’s successful socialization, encompassing their embrace of parents’ and other adults’ values and rules, and responsiveness to the parents during naturalistic interactions, as well as their disregard for rules. We were remarkably successful in recovering the latent structure of children’s positive socialization outcomes, incorporating data from three different sources in both mother- and father-child dyads in FS and mother-child dyads in CAPS. The analyses resulted in meaningful latent variables reflecting overall positive socialization outcomes. In FS, the behavioral measures, children’s reports, and parents’ reports, all assessed twice over the course of preadolescence (at ages 10 and 12) converged meaningfully into a latent construct that encompassed the child’s embrace of adult values, prosociality toward others, and responsive, receptive stance toward the parent, observed in broadly ranging contexts that included highly affectively charged and potentially conflictual topics. As a minor note of caution, internal consistency of one measure – the children’s reports – was relatively modest.

Although, given the significant age difference, the measures of socialization outcomes in CAPS were quite different, we again successfully recovered a meaningful latent construct of successful socialization for mothers and children. It encompassed (low) violations of maternal prohibition, conduct compatible with another adult’s rules, and responsiveness to mother. However, we were unable to recover a parallel latent construct from father-child dyads, due to non-significant correlations between child responsiveness to father and the other outcomes. Unique characteristics of father-child relationships may explain these non-significant correlations (Pacquette, 2004). Bocknek et al. (2017) found that father-child active play, but not other activities, related to preschoolers’ social, cognitive, and emotional outcomes. Thus, children’s behaviors in father-child relationships, including responsiveness, may not be associated with other child socialization behaviors outside of play settings, especially at a young age. The measurement challenges may be part of the reason we failed to support our moderation model in father-child dyads in CAPS.

### Limitations, contributions, and future directions

This work has limitations. The participants in both studies represented low-risk, two-parent families. Racial and ethnic diversity was limited, although in both studies, 20% of families (total *N* = 60) were not “White alone,” and both samples were well representative of our state. Note that from the perspective of replication, demographic comparability of the two samples may be indeed a desired feature of this work.

Upon entry to the studies, the infants were typically developing. Parents were generally gentle and used little power when controlling their children. The children’s IWMs of the parents were overall positive and characterized by relatively high degree of trust and expectations of responsiveness. Children were overall successfully socialized. Nevertheless, we were still able to detect the anticipated detrimental effects of parental power assertion for children with relatively less positive views of their parents.

Consistent with the tenets of developmental psychopathology, future research would benefit from including families representing higher risk levels, or overall broader spectrum of risk. Those risks may include dysfunctional parent-child relationships, especially harsh and punitive discipline, including physical abuse, parents with psychopathology, children with elevated levels of externalizing problems, and family environments characterized by poverty, chaos, stress, lack of support, inter-parental conflict and violence, single parenthood, and other established risk factors.

By the current standards, the sample size in FS, which began two and a half decades ago, was modest, and this limitation must be acknowledged. Nevertheless, we believe that advantages of having two studies supporting an essentially the same developmental process, founded in the same theoretical framework, despite differences in the cohorts, ages, and some measures (a “varied replication,” van IJzendoorn & Bakermans-Kranenburg, 2024) outweigh the concern about the size of the earlier sample.

The data from CAPS were subject to limitations due to the concurrent measurement of power assertion and socialization measures. Our motivation was to match the timing of the assessment of control to that in FS, at age 4.5 years; but age 4.5 was also the final assessment available in the still-ongoing CAPS. To reduce potential bias, we covaried early child difficulty, one potential source of shared variance. We plan to continue to test our model, including future outcomes, some parallel to FS, at later ages, as the study progresses.

Future research should expand the tested framework to include also children’s early attachment security with the parent, conceptualized and modeled as a predictor of their IWMs, which in turn would be modeled as moderators of the effects of parental power assertion on children’s outcomes. In the interest of transparency, we note that we have initially conducted those analyses, and supported such a more complete model in FS, but not in CAPS (the other effects were unchanged). However, given the modest sample size in FS, we decided not to retain early security in the final report.

This work contributes to our understanding of the powerful role children’s representations of their parents may play in determining multifinality of socialization trajectories. Pioneering studies by Bosmans and colleagues, using experimental designs, has demonstrated that in middle childhood, children’s IWMs of parents can be modifiable, leading to increased trust in the caregivers (Bosmans et al., 2019; De Winter et al., 2017, 2018). Consequently, our findings, and more generally, research on children’s mentalization processes, may have translational implications, as those representations may be crucial potential windows for interventions.

## Supplementary Material

1

2

3

4

**Supplementary material.** For supplementary material/s referred to in this article, please visit https://doi.org/10.1017/S0954579425100321.

## Figures and Tables

**Table 1.** Family Study: Descriptive statistics and correlations among study variables

Construct	1	2	3	4	5
1. Parental Power Assertion, Age 4.5 Years	.53***	−.10	−.33**	−.17	−.36**

2. Child IWM of Parent, Age 8 Years	−.06	.67***	.21	−.02	.14

3. Child Internalization of Adult Values, Ages 10 – 12 Years	−.27*	.21	–	.43***	.45***

4. Child Prosocial Behavior, Ages 10 – 12 Years	−.39***	.07	.29*	.50***	.25*

5. Child Responsiveness to Parent, Ages 10 – 12 Years	−.43***	.28*	.53***	.41***	.75***

*M*	0.00	52.10	3.71	2.41	4.90
	0.00	50.58		2.37	5.02

*SD*	0.82	5.66	0.24	0.33	0.77
	0.84	6.16		0.34	0.66

*N*	98	86	82	83	81
	98	84		80	77

*p*	–	.007	–	.129	.067

*Note.* IWM = Internal Working Model. Correlations for mother-child dyads are above the diagonal, and correlations for father-child dyads are below the diagonal. Values on the diagonal represent correlations for the variables across mother-child dyads and father-child dyads. Upper and lower values for mean, standard deviation, and *N* refer to mother-child and father-child dyads respectively. The *p*-values refer to the differences between mother- and father-child dyads where applicable (not applicable to the composites of standardized scores or to child internalization of adult values, unrelated to either dyad).

* *p* < .05.

** *p* < .01.

*** *p* < .001.

**Table 2.** Children and Parents Study: Descriptive statistics and correlations among study variables

Construct	1	2	3	4	5
1. Parental Power Assertion, Age 4.5 Years	.44***	−.17*	.25**	−.26**	−.18*

2. Child IWM of Parent, Age 4.5 Years	−.10	.46***	.09	.11	.07

3. Child Violation of Parental Prohibition, Age 4.5 Years	.26**	−.03	.50***	−.41***	−.40***

4. Child Rule-Compatible Conduct, Age 4.5 Years	−.12	.11	−.37***	–	.25**

5. Child Responsiveness to Parent, Age 4.5 Years	.01	.09	−.07	.17*	.21*

*M*	0.00	0.00	0.08	0.00	19.90
	0.00	0.00	0.05		19.46

*SD*	0.84	0.92	0.18	0.88	2.36
	0.77	0.91	0.13		2.67

*N*	156	153	155	150	156
	147	143	145		147

*p*	–	–	.168	–	.046

*Note.* IWM = Internal Working Model. Correlations for mother-child dyads are above the diagonal, and correlations for father-child dyads are below the diagonal. Values on the diagonal represent correlations for the variables across mother-child dyads and father-child dyads. Upper and lower values for mean, standard deviation, and *N* refer to mother-child and father-child dyads respectively. The *p*-values refer to the differences between mother- and father-child dyads where applicable (not applicable to the composites of standardized scores or to child rule-compatible conduct, unrelated to either dyad).

* *p* < .05.

** *p* < .01.

*** *p* < .001.

## Data Availability

Analyses were not pre-registered. For syntax and coding manuals, please contact the corresponding author. We are unable to share the data for individual families. The participating parents signed consent forms that clearly precluded any sharing of individual data, even if de-identified. We are ethically and legally bound to follow this agreement.
