# Supplementary material for: Children’s representations of parents account for multifinality in outcomes of parental control: Evidence from two studies
Source: Dev Psychopathol. Author manuscript; Available in PMC 2026 Jan 2. (PMC12332769; doi:10.1017/S0954579425100321)
Supplement: 2 [file NIHMS2088560-supplement-2.docx]

Children’s representations of parents account for multifinality in outcomes of parental control: Evidence from two studies

Haley M. Herbert, Juyoung Kim, Grazyna Kochanska

Supplement 2: FS – Coding and Data Aggregation for Selected Constructs

(Contact the authors for complete coding manuals)

***Mothers’ and Fathers’ Power-Assertive Control, Age 4.5 Years***

The parent’s control was coded for every 30-s segment (throughout the entire toy cleanup and whenever the child became involved with the off-limits objects). For each segment, the coder assigned one of the global ratings: *no interaction*, *social exchange*, *gentle guidance*, *control*, and *forceful, negative control* (reliability, kappas, were .94 for “Do”, .76 for “Don’t”). For each segment, the coder also noted the parent’s physical techniques: *physical assertive* (holding the child’s hand firmly, physically preventing the child from leaving the chore, blocking access to toys), and *physical forceful* (taking away a toy abruptly, handling the child roughly). Reliability, kappas, were .83 for “Do” and .68 for “Don’t”).

For each context (“Do” and “Don’t”), each code was tallied and divided by the number of segments, and then weighted, to reflect the amount of used power: -2 = *no interaction*, -1 = *social exchange*, 1 = *gentle guidance*, 2 = *control*, 3 = *forceful control*, 4 = *physical assertive*, and 5 = *physical forceful*. Those figures were summed into weighted power assertion composites for “Do” and for “Don’t”. For “Do”, the scores for mothers were not significantly different from fathers: Mothers, *M* = 1.01, *SD* = 0.40, fathers, *M* = 1.05, *SD* = 0.60. For “Don’t”, mothers’ scores were higher than fathers’, mothers, *M* = -0.61, *SD* = 0.50, fathers, *M* = -0.76, *SD* = 0.48, *t*(96) = 3.30, *p* = .001.

The “Do” and “Don’t” composites cohered, *r*(98) = .35 for mothers, and *r*(98) = .40, for fathers, both *p*s < .001. Those two composites were then standardized and averaged across “Do” and “Don’t”, into one overall power assertion score for each parent.

***Children’s Observed Responsiveness to Parents, Ages 10 and 12***

For each context, coders assigned a responsiveness score to the child, ranging from 1 = *highly unresponsive* to 7 = *highly responsive*. The rating incorporated the child’s sensitivity (detection, interpretation, and prompt, appropriate, and contingent response to the parent’s cues, signals, or overtures, etc.), acceptance (warmth, enjoyment, affection, resentment toward the parent), and cooperation (respect for the parent, acknowledging his/her attempts). Generally, high scores denoted instances when the child’s behavior was likely to please the parent. Reliability, weighted kappas, ranged from .75 to .86 at age 10, and from .74 to .91 at age 12.

The scores cohered across the contexts. Cronbach’s alphas at age 10 were (mother-child dyads first, father-child dyads next) .91 and .94, and at age 12, .93 and .92. The scores were then averaged into one score for the child’s responsiveness to the mother and one to the father; they correlated across ages 10 and 12, *r*(70) = .76, and *r*(68) = .64, respectively, both *p*s < .001. and were averaged across both ages, for the child with each parent.
