# Supplementary material for: Children’s representations of parents account for multifinality in outcomes of parental control: Evidence from two studies
Source: Dev Psychopathol. Author manuscript; Available in PMC 2026 Jan 2. (PMC12332769; doi:10.1017/S0954579425100321)
Supplement: 4 [file NIHMS2088560-supplement-4.docx]

Children’s representations of parents account for multifinality in outcomes of parental control: Evidence from two studies

Haley M. Herbert, Juyoung Kim, Grazyna Kochanska

Supplement 4: CAPS – Coding and Data Aggregation for Selected Constructs

(Contact the authors for complete coding manuals)

***Mothers’ and Fathers’ Power-Assertive Control, Age 4.5 Years***

For each segment, coders selected one of four mutually exclusive codes: *no control*, *gentle guidance* (parent directs child behavior in a subtle, gentle, manner), *control* (parent is assertive, adopts a matter-of-fact, means-business manner; some pressure present), or *power-assertive, negative control* (parent may raise voice, appear angry, punitive, or use threats). Reliability, weighted kappas, ranged from .61 to .92.

The occurrences of each code were tallied (separately for the cleanup and other interactions; due to slight variation in duration in the latter, we divided those tallies by the number of segments). Those figures were weighted to reflect the amount of parental pressure: *no control* by 1, *gentle control* by 2, *control* by 3, and *power-assertive, negative control* by 4. Those numbers were summed into power-assertive composites (separately for the cleanup and other interactions). Those two scores correlated, mothers, *r*(156) = .42, *p* < .001, and fathers, *r*(147) = .19, *p* = .022; they were standardized and averaged into overall power assertion for each parent. Mothers’ and fathers’ scores in the cleanup context were not significantly different: Mothers, *M* = 41.58, *SD* = 5.15, fathers, *M* = 41.49, *SD* = 6.28. In the other interactions, mothers’ scores were higher than fathers’, mothers, *M* = 1.42, *SD* = 0.18, fathers, *M* = 1.38, *SD* = 0.17, *t*(145) = 2.76, *p* = .006.

#### **Children’s IWMs of Parents, Age 4.5 Years**

We used two codes (each given to the entire story): Good Representation of the parent and Comfort offered by the parent. Good Representation reflected the child’s overall description of the parent who, in response to the child’s distress, was depicted as**protective, affectionate, forgiving, helpful, warm, composed,**and emotionally present; understanding the child’s feelings, empathic and reassuring; resourceful, trustworthy, and competent. The parent successfully comforted the child, assuaged child distress, and helped the child return to play.

Coders assigned a score from 0 to 4: 0 = *no evidence*, 1= *some evidence present,* 2 = *clear evidence present,* 3 = *strong, somewhat consistent****, detailed evidence present,* 4 = *rich, abundant evidence present*. Each story also received a Comfort score for evidence of the parent as a source of comfort:** 0 = *absent*, 1 = *present* (affection, e.g., “Mom will kiss him”; protection, e.g., “He’ll say don’t worry, spider is gone”). Reliability, weighted kappas, were .81 to .92

We then averaged the scores of Good Representations and Comforting regarding the parent across the three stories. Children’s perceptions of mothers and fathers were not significantly different, either for Good Representations, mothers, *M* = 2.22, *SD* = 1.01, fathers, *M* = 2.16, *SD* = 0.89, or Comforting, mothers, *M* = 0.56, *SD* = 0.37, fathers, *M* = 0.53, *SD* = 0.34. Not surprisingly, the two scores correlated; for children’s perceptions of mothers, *r*(153) = .70, *p* < .001, and of fathers, *r*(143) = .67, *p* < .001. They were then standardized and aggregated into children’s positive representation (of each parent).

***Children’s Observed Rule-Compatible Conduct, Age 4.5 Years***

For every 3-s segment, we coded child behavior as rule compatible or else, violating one or more rules. The codes were tallied and divided by the number of segments when the child was involved in the game (occasionally, children disengaged and stopped playing). Reliability, kappas, were .78 – 1.00. The (reversed) latencies to each type of violation were also averaged. The final composite of rule-compatible conduct included the rule-compatible behavior, the (reversed) score of averaged rule-violating behavior, and (reversed) score of averaged latencies to each violation (after standardizing each variable).
